# ω-3 and ω-6 Fatty Acids Modulate Conventional and Atypical Protein Kinase C Activities in a Brain Fatty Acid Binding Protein Dependent Manner in Glioblastoma Multiforme

**DOI:** 10.3390/nu10040454

**Published:** 2018-04-06

**Authors:** Marwa E. Elsherbiny, Hua Chen, Marwan Emara, Roseline Godbout

**Affiliations:** 1 Department of Pharmacology and Toxicology, Ahram Canadian University, 6th of October City, Giza 12566, Egypt; 2 Department of Oncology, Cross Cancer Institute, University of Alberta, Edmonton, AB T6G 1Z2, Canada; Hua.Chen@albertahealthservices.ca; 3 Center for Aging and Associated Diseases, Zewail City of Science and Technology, Giza 12578, Egypt; memara@zewailcity.edu.eg

**Keywords:** Glioblastoma multiforme, brain fatty acid binding protein, protein kinase C, docosahexaenoic acid, arachidonic acid

## Abstract

Glioblastoma multiforme (GBM) is a highly infiltrative brain cancer with a dismal prognosis. High levels of brain fatty acid binding protein (B-FABP) are associated with increased migration/infiltration in GBM cells, with a high ratio of arachidonic acid (AA) to docosahexaenoic acid (DHA) driving B-FABP-mediated migration. Since several protein kinase Cs (PKCs) are overexpressed in GBM and linked to migration, we explored a possible relationship between B-FABP and levels/activity of different PKCs, as a function of AA and DHA supplementation. We report that ectopic expression of B-FABP in U87 cells alters the levels of several PKCs, particularly PKC*ζ*. Upon analysis of *PKCζ* RNA levels in a panel of GBM cell lines and patient-derived GBM neurospheres, we observed a trend towards moderate positive correlation (*r* = 0.624, *p* = 0.054) between B-FABP and *PKCζ* RNA levels. Analysis of PKC activity in U87 GBM cells revealed decreased typical PKC activity (23.4%) in B-FABP-expressing cells compared with nonexpressing cells, with no difference in novel and atypical PKC activities. AA and DHA modulated both conventional and atypical PKC activities in a B-FABP-dependent manner, but had no effect on novel PKC activity. These results suggest that conventional and atypical PKCs are potential downstream effectors of B-FABP/fatty acid-mediated alterations in GBM growth properties.

## 1. Introduction

Glioblastoma multiforme (GBM) accounts for ~70% of malignant primary brain tumors and is associated with poor prognosis with 2- and 5-year survival rates of 26–33% and 4–5%, respectively [1,2]. Despite aggressive treatment, almost all GBMs recur, with recurrence associated with tumor infiltrative properties and poor response to treatment [3,4].

Factors that contribute to GBM infiltration include brain fatty acid binding protein (B-FABP) and protein kinase C (PKC) [5,6,7]. FABPs in general are thought to mediate the physiological and pathological actions of different polyunsaturated fatty acids (PUFAs) by facilitating their intracellular trafficking [8,9]. B-FABP in particular is expressed in the developing brain [10,11] as well as in GBM tumors [12,13] where it is expressed in areas of tumor infiltration and has been identified as a marker of poor prognosis. B-FABP binds ω-3 and ω-6 PUFAs, with a higher affinity reported for ω-3 PUFAs such as docosahexaenoic acid (DHA) [8,14]. In the developing brain, B-FABP is expressed in radial glial cells that establish the radial glial fiber system along which neurons migrate [10,15]. In GBM cell lines, B-FABP modulates cell migration in a fatty-acid-dependent manner [6]. Changing the ratio of DHA/arachidonic acid (AA) in the culture medium has a profound effect on GBM cell migration, with an increased AA/DHA ratio associated with increased migration, and an increased DHA/AA ratio associated with reduced migration [6]. Based on these data, it was postulated that the lipid environment together with B-FABP expression are determinants of GBM infiltration [6,16]. While not demonstrated in GBM, AA and DHA also were shown to modulate the activities of different PKCs in breast cancer cell lines [17].

PKC is an intracellular effector molecule that regulates cell proliferation, differentiation, and migration [18]. PKCs are classified into three subclasses: conventional (also called typical), novel, and atypical [18,19]. Conventional PKCs are calcium dependent and are activated by diacylglycerol (DAG). Novel PKCs do not require calcium for activation but are stimulated by DAG. Atypical PKCs require neither calcium nor DAG for activation. Roles of different PKCs in cancer progression have been extensively studied [20,21] with some studies showing synergistic anticancer effects for PKC inhibitors when combined with radiation therapy or tyrosine kinase inhibitors [22,23].

B-FABP expression is up-regulated in GBM compared with normal brain and low-grade astrocytomas [12,24], with increased expression being associated with tumor infiltration and worse prognosis [25,26]. Several PKCs are overexpressed in GBM cell lines and GBM-derived stemlike cells with links to cell migration and invasion [27,28,29,30]. Here, we explore the possibility that PKCs are B-FABP targets. We report that ectopic B-FABP expression in U87 GBM cells results in alterations in the expression of PKCs, with most changes being relatively minor except for the *PKCζ* gene. B-FABP expression negatively modulates conventional PKC activity, but has no effect on novel or atypical PKC activities. In addition, we found that AA and DHA modulate conventional and atypical PKC activities in a B-FABP-dependent manner, with AA and DHA restoring B-FABP-mediated reduction in conventional PKC activity, and DHA activating atypical PKC activity only in the presence of B-FABP. 

## 2. Materials and Methods

### 2.1. Cell Culture and Reverse Transcription PCR (RT-PCR) Analysis

Levels of PKC RNAs in U87 control versus B-FABP-expressing cells were assessed based on gene expression microarray analysis. Of note, B-FABP levels in our U87 B-FABP-positive cultures are similar to those found in GBM cells that naturally express B-FABP [31]. Agilent microchip arrays (44K format), printed with 60-mer oligonucleotides, were used for this experiment. RNAs were prepared from three independent cultures of U87 GBM pREP4 control cells and three independent cultures of U87 pREP4-B-FABP transfected cells [31] using the QIAGEN RNAPlus kit. These six RNA preparations were labeled with Cy3-UTP and used for microchip array hybridization. Relative gene expression levels were normalized to account for hybridization variation across and between microchips. Semiquantitative RT-PCR was used to verify differential expression of selected PKCs in U87 control and B-FABP-transfected cells as well as in GBM cell lines that are either negative (T98, U87, A172, CLA, and M021) or positive (U251, U373, M049, M103, and M016) for B-FABP [12,31,32]. Sources of GBM cell lines have been described previously [33] and they are as follows: T98, from Walter Nelson-Rees, Naval Biomedical Research Station, Oakland, CA, USA; U87, U251, and U373, from Jorgen Fogh, Sloane Kettering Institute, Rye, NY, USA; A172, from Stuart A. Aaronson, NCI, Bethesda, MD, USA; CLA, from Paul Kornblith, Albert Einstein College of Medicine, Montefiore Medical Center, Bronx, NY, USA. M016, M021, M049, and M103 GBM cell lines were obtained from Drs. M. Joan Allalunis-Turner and Rufus S. Day III. These cell lines were established from biopsies obtained with patient consent prior to surgery. All procedures were approved by the Alberta Cancer Board Ethics Committee on Human Experimentation. Cells were cultured in Dulbecco’s modified essential medium (DMEM) supplemented with 10% fetal calf serum, 100 μg/mL streptomycin, and 100 units/mL penicillin, and maintained at 37 °C in a humidified 5% CO_2_ atmosphere. Five GBM tumor neurosphere cultures (ED501, ED502, ED509, ED511, and ED512) were generated from GBM patients, with informed consent from patients enrolled in the study, including review of an information package with a study nurse and physician and signing of a detailed consent form. The protocol was approved by the University of Alberta Research Ethics Board and the Government of Canada Health Protection Branch (IND # 056621). These cells were cultured in DMEM/F12 supplemented with human epidermal growth factor (20 ng/mL; PeproTech, Montreal, QC, Canada), human basic fibroblast growth factor (10 ng/mL; Cedarlane, Burlington, ON, Canada), and 0.5X B27 supplement (Thermo Fisher Scientific, Waltham, MA, USA). 

Total RNA was isolated from cell lines and neurosphere cultures using TRIzol Reagent (Invitrogen, Carlsbad, CA, USA) according to the manufacturer’s protocol and quantified by measuring the absorbance at 260 nm. First strand cDNA was generated using SuperScriptTM II Reverse Transcriptase (Invitrogen) using the manufacturer’s protocol. The resulting cDNAs were subjected to PCR amplification; products were separated in a 1% agarose gel, stained using ethidium bromide, and visualized under UV light. The following primers were used for RT-PCR: B-FABP, forward 5′-TGG AGG CTT TCT GTG CTA C-3′, reverse 5′-TAG GAT AGC ACT GAG ACT TG-3′; PKCζ, forward 5′-CAG GAG AAG AAA GCC GAA TC-3′, reverse 5′-CAT CCA TCC CAT CGA TAA CTG-3′; β-actin, forward 5’-CTG GCA CCA CAC CTT CTA C-3′, reverse 5′-CAT ACT CCT GCT TGC TGA TC-3′. Number of cycles used for PCR amplification was 32 for B-FABP, 32 for PKCζ, and 28 for β-actin.

### 2.2. Preparation of Fatty Acids

Fatty acids (Sigma, St-Louis, MO, USA) were dissolved in ethanol, then complexed to fatty-acid-free bovine serum albumin (BSA) (Roche Applied Science, Penzberg, Germany) over a steady stream of nitrogen gas. Fatty acids were conjugated at a fatty acid/BSA molar ratio of 3:1 [34]. The final concentration of ethanol in our experiments was <0.001%. Cells were cultured to ~70% confluency then incubated for 24 h with 60 μM BSA-conjugated DHA or AA or BSA alone.

### 2.3. Protein Kinase C Activity

Cell lysates for kinase activity were prepared as described [35]. Briefly, cells were washed twice with ice-cold PBS then incubated in lysis buffer (50 mM HEPES (N-2-hydroxyethylpiperazine-N′-2-ethanesulfonic acid, pH 7.4), 100 mM NaCl, 50 mM sodium fluoride, 5 mM β-glycerophosphate, 2 mM EDTA, 2 mM EGTA (ethylene glycol-bis(β-aminoethylether)-N,N,N,N-tetraacetic acid), 1 mM sodium vanadate, 1% Nonidet P-40, 1 mM PMSF, 1X protease inhibitor cocktail (Roche)) for 10 min at 4 °C. Lysates were then microcentrifuged for 10 min at maximum speed and the supernatant was collected and stored at −80 °C. The protein concentration of cell lysates was measured using the Bradford reagent.

Histone phosphorylation was assayed in the presence of appropriate co-factors to determine PKC class-specific activity. The assay mixture (total volume 20 µL) contained reaction buffer (20 mM Tris-HCl pH 7.5, 5 mM MgCl_2_, 200 µM CaCl_2_), 100 µg/mL histone from calf thymus (Sigma), 5 µM cold ATP (mixed with γ^32^P-ATP for a final concentration of 2 µCi), and 20 µg/mL phosphatidylserine (Sigma). Diolein (2 µg/mL) was incorporated in the mixtures used for assaying conventional and novel activities. CaCl_2_ was excluded and 2 mM EGTA was added when novel and atypical activities were assayed [36]. The reaction was started by adding 2 µg lysates and was allowed to proceed for 5 min at 30 °C. Phosphorylated proteins were separated by SDS-PAGE and dried gels were exposed to X-ray film [37].

### 2.4. Statistical Analysis

Data are reported as mean ± standard error of the mean (SEM) of 3–6 experimental replicates per group. In analyzing differences in PKC expression, unpaired Student’s *t*-test was used to assess the significance of differences between groups. Two-way analysis of variance was used to assess the interaction between treatments (AA, DHA, and BSA) and B-FABP expression status (control U87 vs B-FABP-expressing U87 cells) in terms of class-specific PKC activity. Microsoft Excel (Microsoft, Redmond, WA, USA) and SigmaPlot 12.0 (Systat software, Inc. Chicago, IL, USA) were used in the statistical analysis. The level of significance was set at *p* < 0.05.

## 3. Results

**Figure 1:**
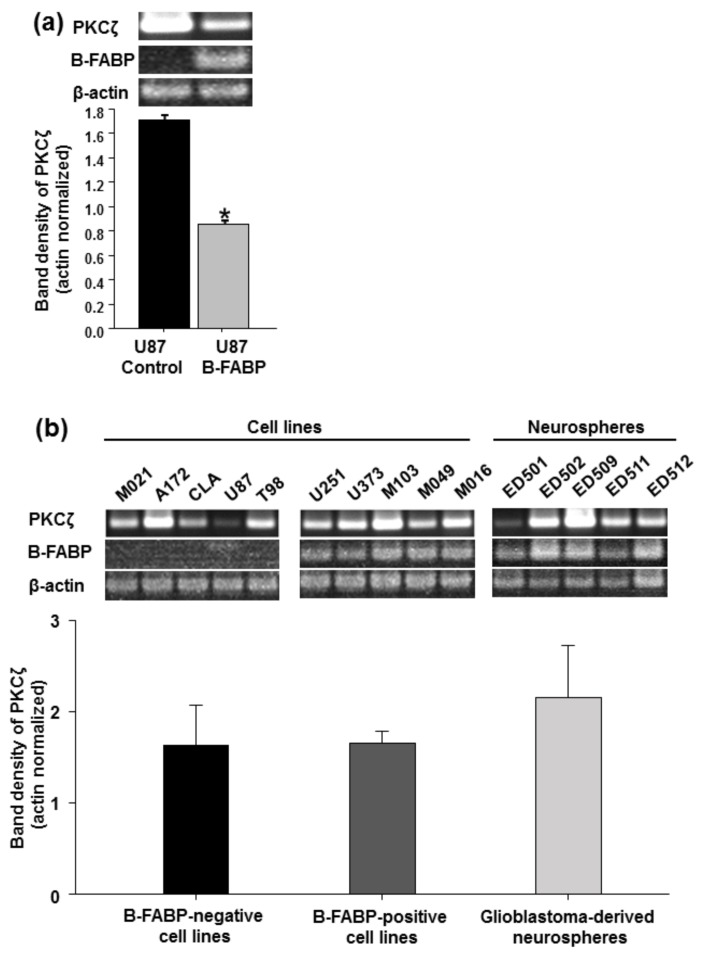
B-FABP and PKCζ expression in glioblastoma multiforme (GBM) cell lines and neurospheres: (**a**) *PKCζ* and *B-FABP* expression in U87 GBM cells stably transfected with either empty pREP4 vector (U87 Control) or a pREP4-FABP7 expression construct (U87 B-FABP), *n* = 3 independent replicates and * indicates significant difference compared with U87 Control cells; (**b**) *PKCζ* and *B-FABP* expression in GBM cell lines that are negative for B-FABP (M021, A172, CLA, U87, and T98), GBM cell lines that are positive for B-FABP (U251, U373, M103, M049, and M016), and GBM tumor neurosphere cultures generated from five different GBM patients (ED501, ED502, ED509, ED511, and ED512). Band densities were obtained by densitometric scanning of each band using Adobe Photoshop CS4.

An initial survey of *PKC* RNA levels in stably transfected U87 GBM control cells (B-FABP-negative) versus stably transfected U87 B-FABP (B-FABP-positive) cells [31] was carried out using cDNA gene expression microarray data. B-FABP expression in U87 cells affected the RNA levels of several *PKC* genes (Table 1). For example, *PKCα*, *δ*, and *η* were upregulated 1.6-, 1.6-, and 1.89-fold, respectively, whereas *PKCε*, *ι*, and *ζ* were downregulated 3-, 1.8-, and 25-fold, respectively, compared with U87 control cells. *PKCζ* showed the most pronounced change in gene expression as a result of B-FABP expression. Therefore, we studied this gene further by semiquantitative RT-PCR analysis. In general agreement with our microarray data, ectopic B-FABP expression in U87 cells was associated with a reduction in *PKCζ* RNA levels (Figure 1a).

**Figure 2:**
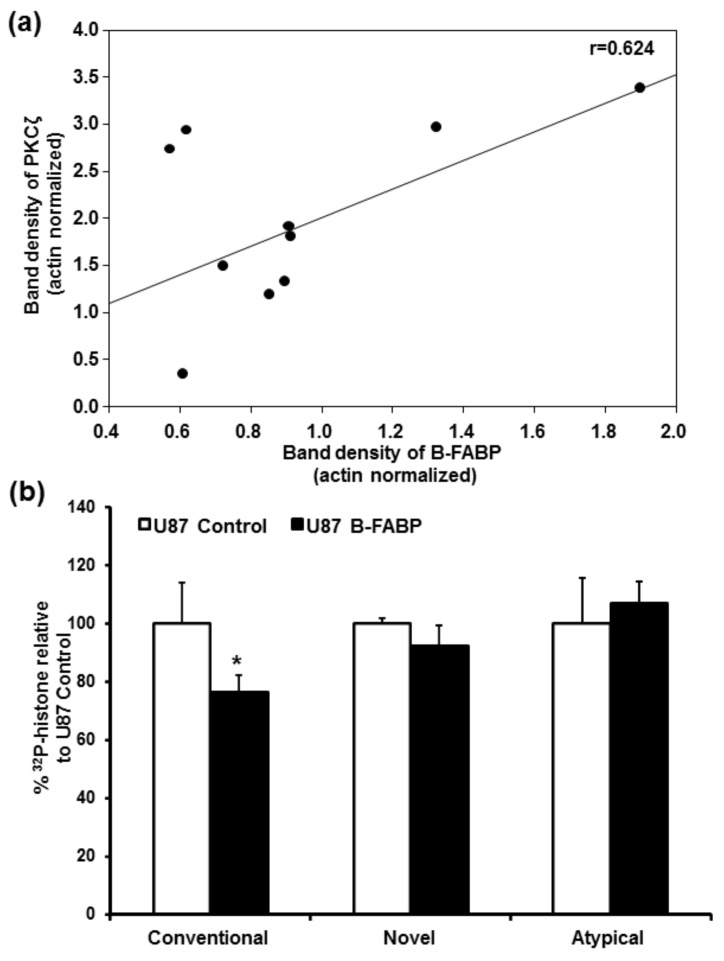
(**a**) Correlation between *B-FABP* and *PKCζ* RNA levels in five B-FABP-positive GBM cell lines (U251, U373, M103, M049, and M016) and in five GBM tumor neurosphere cultures derived from different GBM patients (ED501, ED502, ED509, ED511, and ED512). *PKCζ* RNA levels are indicated on the *y* axis and *B-FABP* RNA levels are indicated on the *x* axis. Band densities were obtained by densitometric scanning of each band using Adobe Photoshop CS4; (**b**) B-FABP is associated with reduced conventional PKC activity but has no effect on novel and atypical PKC activities. Class-specific PKC activity, calculated as percentage of ^32^P-histone relative to U87 control cells, in U87 cells transfected with either empty pREP4 vector (U87 Control) or a pREP4-FABP7 expression construct (U87 B-FABP).

Next, we examined *PKCζ* expression in a panel of 15 GBM lines: 5 B-FABP-negative, 5 B-FABP-positive, and 5 B-FABP-positive tumor neurospheres derived from GBM patients. There was no significant difference in *PKCζ* RNA levels between the 5 B-FABP-negative GBM cell lines and 5 B-FABP-positive GBM lines (Figure 1b). Furthermore, all five GBM-derived tumor neurosphere cultures co-expressed *B-FABP* and *PKCζ* (Figure 1b). When we assessed the correlation between *B-FABP* and *PKCζ* expression in B-FABP-positive cell lines and neurosphere samples (*n* = 10), we observed a trend towards positive correlation for the expression of both genes with the Pearson Correlation Coefficient (*r*) being 0.624, *p* = 0.054 (Figure 2a). These results are in contrast to our findings in U87 cells ectopically expressing B-FABP, which showed reduced *PKCζ* expression compared with control (B-FABP-negative) U87 cells. 

In light of the extensive heterogeneity associated with GBM tumors and cell lines, combined with the difficulty associated with extrapolating findings from mRNA expression studies to protein expression and activity [38,39], we decided to use our paired isogenic U87 lines (B-FABP negative and B-FABP positive) to further examine a possible relationship between B-FABP expression and PKC activity. Conventional PKC activity was significantly reduced (by 23.4%) in B-FABP-expressing U87 cells compared with U87 control cells (*p* = 0.014), with no change observed in either novel or atypical PKC activity (Figure 2b). Thus, the activity data showing changes in conventional PKC activity but not in atypical PKC activity, combined with gene expression analysis showing little change in conventional PKC RNA levels but a significant reduction in atypical PKC*ζ* RNA levels, suggest that additional factors, including post-transcriptional mechanisms, may be regulating different PKCs irrespective of B-FABP expression, with B-FABP expression only affecting conventional PKC activity.

**Figure 3:**
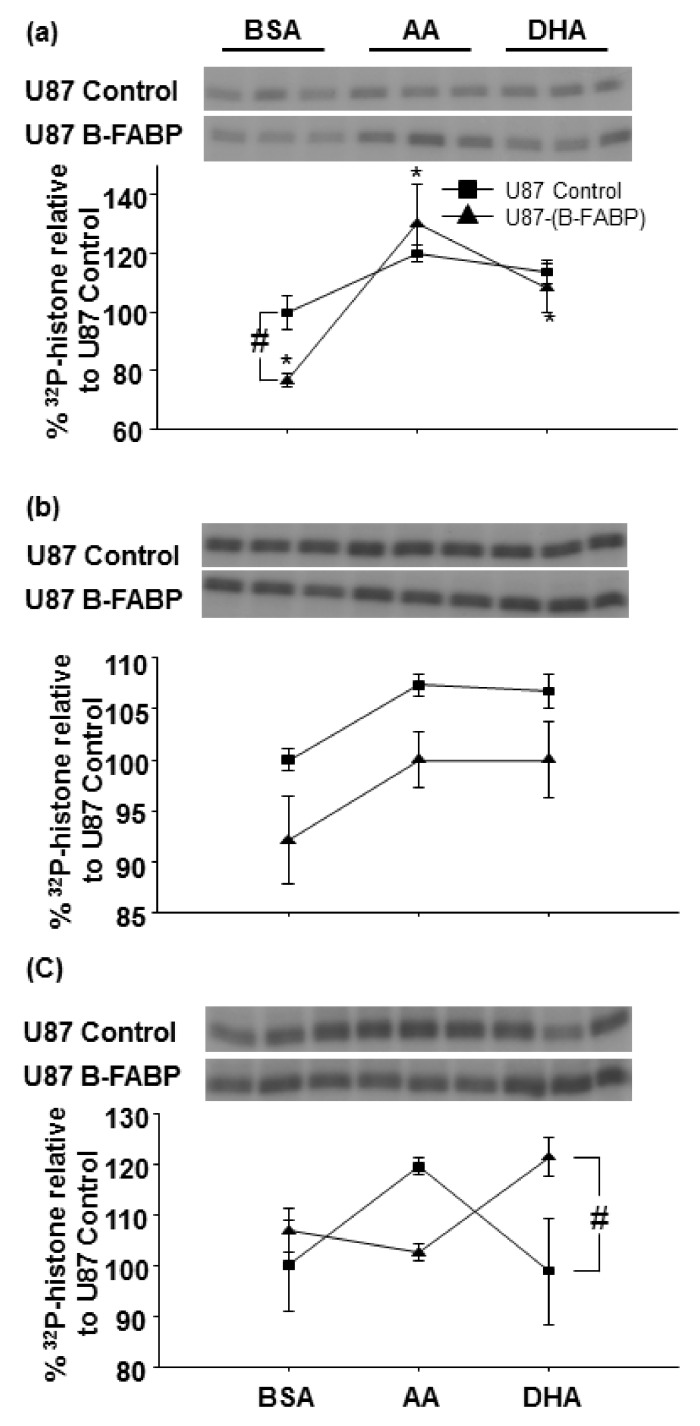
Arachidonic acid (AA) and docosahexaenoic acid (DHA) modulate conventional and atypical PKC activities in U87 cells expressing B-FABP. Two-way effect plot of least square mean PKC activity (*y* axis) calculated as percentage of U87 control BSA-treated cells vs treatment (*x* axis) for (**a**) conventional PKC activity; (**b**) novel PKC activity; and (**c**) atypical PKC activity measured in U87 cells transfected with either empty pREP4 vector (U87 Control) or a pREP4-FABP7 expression construct (U87 B-FABP). Cells were treated with 60 µM BSA, 60 µM AA, or 60 µM DHA (*n* = 3–6/treatment). ^#^ indicates differences relative to the U87 control from the corresponding treatment group and * indicates a difference in the effect of the treatment for the same cell type.

Based on literature showing that B-FABP and PKC activities are modulated by ω-3 and ω-6 fatty acids, we next compared the activities of different PKC subclasses in U87 control and B-FABP-expressing cells that were treated with either BSA, AA, or DHA (Figure 3a–c). Conditions for this assay were based on preliminary time, protein, histone, and ATP linearity studies. Two-way ANOVA was used to test effects of treatment (BSA vs AA vs DHA) and B-FABP expression (U87 control vs U87 B-FABP) on class-specific PKC activity. The interaction between the two factors was also assessed. Based on this analysis, a significant interaction between fatty acid treatment and B-FABP expression was observed for conventional and atypical PKC activities (Figure 3a, c, *p* = 0.048 and 0.025, respectively). As for the novel PKC activity, there was no significant interaction between fatty acid treatment and B-FABP expression (Figure 3b, *p* = 0.976).

Interestingly, fatty acid treatment did not significantly alter conventional PKC activity in U87 control cells; however, conventional PKC activity was significantly increased in B-FABP-expressing cells, with a greater increase in activity observed in AA- (by 53.9%) compared with DHA-treated cells (by 31.6%) (Figure 3a). The fact that neither DHA nor AA affected PKC activity in U87 control cells suggests that this increase in activity may be mediated by B-FABP. 

Although two-way ANOVA showed an overall significant effect for fatty acid treatment (*p* = 0.027) and B-FABP expression (*p* = 0.007) on novel PKC activity, pairwise comparisons within each fatty acid treatment group did not reach statistical significance. A trend towards lower novel PKC activity in BSA-, AA-, and DHA-treated B-FABP-expressing U87 cells compared with their respective controls was observed with *p* values of 0.064, 0.084, and 0.110, respectively (Figure 3b). However, the differences were minor. 

As for the atypical PKC activity, there was a statistically significant interaction between fatty acid treatment and B-FABP (*p* = 0.025). B-FABP-expressing cells showed higher (by 22.5%) activity when treated with DHA compared to control cells (*p* = 0.025, Figure 3c). We also observed a trend towards increased activity in AA-treated U87 control cells compared with AA-treated B-FABP-expressing U87 cells (*p* = 0.078). Thus, AA and DHA appear to have opposite effects on atypical PKC activity depending on whether B-FABP is expressed or not.

## 4. Discussion

B-FABP and several PKCs share common functional features such as modulation of activity by ω-3 and ω-6 PUFAs and involvement in cancer cell growth and migration [5,6,28,38,39,40]. To investigate a possible relationship between B-FABP and PKC in GBM, we examined PKC gene expression and activity as a function of B-FABP expression in the U87 GBM cell line. In addition, we assessed the influence of two B-FABP ligands—AA and DHA—on class-specific PKC activity and its dependence on B-FABP. We report that ectopic expression of B-FABP results in variations in the expression of multiple *PKCs* with the changes being mostly minor except for *PKCζ.* In contrast to its effect on *PKCζ* RNA levels, B-FABP expression was correlated with reduced conventional PKC activity without affecting novel or atypical PKC activities. Our results show that AA and DHA modulate conventional and atypical PKC activities in a B-FABP-dependent manner, with AA and DHA restoring B-FABP-mediated reduction in conventional PKC activity and DHA activating atypical PKC activity only in the presence of B-FABP. In contrast, AA appears to activate atypical PKC activity only in the absence of B-FABP.

Although variations in the gene expression profiles of several *PKC* genes were observed in response to *B-FABP* expression in U87 cells, the only PKC that was strongly affected was *PKCζ*. However, we found no difference in *PKCζ* RNA levels between GBM cell lines that naturally express or do not express B-FABP. These data indicate that B-FABP by itself does not regulate *PKCζ* gene expression. These results are not surprising in light of the cellular and molecular heterogeneity observed within GBM tumors and across GBM cell lines. Thus, the growth properties of GBM cell lines may be controlled through a variety of mechanisms. For this reason, we chose to focus our analyses on a matched pair of GBM cells: U87 control cells negative for B-FABP and U87 cells expressing ectopic B-FABP at physiologically relevant levels. Using this model, we found that B-FABP significantly modulates fatty-acid-dependent activation of conventional and atypical PKC activities in the U87 GBM cell line. Indeed, DHA treatment increased atypical PKC activity, but only in the presence of B-FABP. In contrast, AA appears to increase atypical PKC activity, but only in the absence of B-FABP (Figure 3c). Furthermore, the reduction in conventional PKC activity observed in B-FABP-expressing U87 cells was restored by the addition of AA and to a lesser extent by DHA (Figure 3a). The significance of these findings in terms of cell function has yet to be determined, but may have implications for GBM cell migration, tumor infiltration, and resistance to various treatment modalities. 

Besides the changes noted in *PKCζ* gene expression, we observed changes in the expression of other *PKC* genes belonging to the conventional and the novel PKC subclasses. However, these changes were relatively minor (at most threefold) (Table 1) and did not translate into equivalent changes in class-specific activity except for conventional PKC activity (Figure 2b) which was decreased in response to B-FABP expression. This is in contrast to the overall 1.6-fold increase observed in *PKCα* gene expression. Again, this observation highlights the possibility that other mechanisms, including post-transcriptional mechanisms, likely regulate the expression and/or function of different PKCs in GBM cells. It is worth mentioning that it has been shown that B-FABP expression in the U87 cell line significantly reduces its proliferation potential [32], with similar results being reported for renal carcinoma cell lines [41]. As PKCα, a conventional PKC, is well recognized for its role in GBM cell proliferation [37], our results suggest that the inhibitory effect of B-FABP on GBM proliferation is potentially mediated by inhibition of PKCα as suggested by the decrease in conventional activity (Figure 2b).

Some novel PKC genes such as PKCδ [28] and ε [42,43] were shown to be involved in GBM migration and invasion whereas PKCη [44] is associated with cell proliferation. To our knowledge, the effects of ω-3 and ω-6 fatty acids on novel PKCs have not been explored in GBM. Here, we show that AA and DHA do not significantly alter overall novel PKC activity in U87 cells, with no change being observed when B-FABP is ectopically expressed. This is in contrast to normal dog cardiomyocytes, in which a fish-oil-rich diet was shown to decrease PKCδ and ε activation [39]. In addition, AA was shown to activate PKCε and PKCµ in the B-FABP-positive human breast cancer cell line MDA-MB-435, with consequent enhancement of cell adhesion [17]. 

Atypical PKCζ and ι have also been implicated in GBM cell proliferation, migration, and invasion [20,38,45,46] with a report showing up-regulation and down-regulation of PKCι and PKCζ, respectively, in neural stemlike cells derived from primary GBM patients compared with normal brain tissue [27]. The effects of AA and DHA on these PKCs have not been studied in GBM. However, in the human macrophage cell line U937, only the atypical PKCζ was detected. In contrast to our overall atypical PKC activity results, the basal activity of PKCζ, as indicated by its phosphorylation state, was reduced by AA in U938 cells [47]. Another report shows that AA-mediated Ca^2+^ sensitization and phosphorylation of myosin regulatory light chain were inhibited by a pseudo-substrate peptide inhibitor of the atypical PKCs, thus indicating that AA activates atypical PKCs, which is consistent with our results here with U87 GBM cells [48]. DHA, on the other hand, has been shown to up-regulate PKCζ protein expression and atypical PKC activity in guinea pig epidermis which is associated with DHA-induced skin hyperproliferation [49,50]. These observations are consistent with our results showing DHA-mediated activation of atypical PKCs in U87 cells with B-FABP being essential for this activation. Future studies aimed at exploring the interplay between B-FABP, its fatty acid ligands (AA and DHA), and PKCs of the conventional and atypical subclasses, along with examining the effects of this interplay on GBM growth properties, should shed light on pathways responsible for GBM aggressiveness and recurrence.

## 5. Conclusions

We show that the expression of PKCs, particularly PKCζ, is affected by B-FABP expression in U87 GBM cells. Our data suggest that conventional and atypical PKCs may be downstream effectors of B-FABP, with AA and DHA modulating PKC activity. 

## Figures and Tables

**Table 1:** Protein kinase C (PKC) genes that are differentially expressed in the U87 control compared with U87 brain fatty acid binding protein (B-FABP) cell lines based on cDNA microarray data.

Gene	Fold Change with B-FABP Expression	*p*-value ^b^, *n* = 6
**Conventional PKCs**		
PKCα	+1.63	0.001
PKCβ	NC ^a^	0.138
PKCγ	NC ^a^	0.502
**Novel PKCs**		
PKCδ	+1.6	0.0001
PKCε	−3.0	<0.0000001
PKCη	+1.89	0.015
**Atypical PKCs**		
PKCι	−1.8	<0.0000001
PKCζ	−25	<0.0000001

^a^ NC indicates no change, ^b^ *p*-values were calculated using two-tailed Student’s *t*-test.
